# *HLA-G* 14 bp Ins/Del (rs66554220) Variant Is Not Associated with Breast Cancer in Women from Western Mexico

**DOI:** 10.3390/cimb45080432

**Published:** 2023-08-16

**Authors:** Denisse Stephania Becerra-Loaiza, Luisa Fernanda Roldan Flores, Luis Antonio Ochoa-Ramírez, Bricia M. Gutiérrez-Zepeda, Alicia Del Toro-Arreola, Ramón Antonio Franco-Topete, Andrés Morán-Mendoza, Antonio Oceguera-Villanueva, Antonio Topete, David Javalera, Antonio Quintero-Ramos, Adrián Daneri-Navarro

**Affiliations:** 1 Centro Universitario de Ciencias de la Salud, Laboratorio de Inmunología, Departamento de Fisiología, Universidad de Guadalajara, Sierra Mojada #950, Guadalajara 44340, Mexico; 2 Doctorado en Genética Humana, Centro Universitario de Ciencias de la Salud, Departamento de Biología Molecular y Genómica, Universidad de Guadalajara, Sierra Mojada #950, Guadalajara 44340, Mexico; 3 Laboratorio de Medicina Genómica, Hospital General de Culiacán, Culiacán 80230, Mexico; 4 Centro Universitario de Ciencias de la Salud, Laboratorio de Patología, Departamento de Microbiología y Patología, Universidad de Guadalajara, Sierra Mojada #950, Guadalajara 44340, Mexico; 5 Centro Médico Nacional de Occidente, Hospital de Gineco Obstetricia, Instituto Mexicano del Seguro Social, Av. Belisario Domínguez #1000, Guadalajara 44340, Mexico; 6 Instituto Jalisciense de Cancerología, Secretaría de Salud, Coronel Calderón #715, Guadalajara 44280, Mexico; 7 Departamento de Aparatos y Sistemas II, Universidad Autónoma de Guadalajara, Av. Patria #1201, Zapopan 45129, Mexico; 8 Centro Médico Nacional de Occidente, Unidad de Investigación Biomédica 02, Hospital de Especialidades, Instituto Mexicano del Seguro Social, Av. Belisario Domínguez #999, Guadalajara 44340, Mexico

**Keywords:** breast cancer, HLA-G, Mexican population, rs66554220, 14-bp Ins/Del, western Mexico, stage IV, clinical

## Abstract

HLA-G is a physiology and pathologic immunomodulator detrimentally related to cancer. Its gene is heavily transcriptionally and post-transcriptionally regulated by variants located in regulator regions like 3′UTR, being the most studied Ins/Del of 14-bp (rs66554220), which is known to influence the effects of endogen cell factors; nevertheless, the reports are discrepant and controversial. Herein, the relationship of the 14-bp Ins/Del variant (rs66554220) with breast cancer (BC) and its clinical characteristics were analyzed in 182 women with non-familial BC and 221 disease-free women as a reference group. Both groups from western Mexico and sex–age-matched (sm-RG). The rs66554220 variant was amplified by SSP-PCR and the fragments were visualized in polyacrylamide gel electrophoresis. The variant rs66554220 was not associated with BC in our population. However, we suggest the Ins allele as a possible risk factor for developing BC at clinical stage IV (OR = 3.05, 95% CI = 1.16–7.96, *p* = 0.01); nevertheless, given the small stratified sample size (n = 11, statistical power = 41%), this is inconclusive. In conclusion, the 14-bp Ins/Del (rs66554220) variant of *HLA-G* is not associated with BC in the Mexican population, but might be related to advanced breast tumors. Further studies are required.

## 1. Introduction

Breast cancer (BC) is the most common and fatal type of cancer among the female population worldwide [1]. The same is true for Mexico where, in the last three decades, BC incidence and mortality have increased importantly [1]. The risk of developing BC depends on ethnicity, family history and environmental factors, with genetics representing a central element to identify possible biomarkers [2].

Phenotypic heterogeneity in BC shows a considerable challenge for tumor therapy [3]. Recently, Anna et al. evidenced a component from non-classical MHC-I, named HLA-G, as an excellent and effective target for CAR-T immunotherapy [4]. HLA-G constitutive expression is usually restricted to a few human tissues, but its ectopic expression has been demonstrated in different kinds of tumors [5,6]. Moreover, it has been related to disease stage and outcomes, metastatic status and response to different therapies [3]. Nonetheless, it is still difficult to determine a clear correlation between the HLA-G isoforms and the disease features [7].

The *HLA-G* gene is within the *MHC* cluster in 6p21.3, encoding for a non-classical HLA-I molecule with (1) immunosuppressive properties, (2) the capacity for ectopic expression in pathological conditions and (3) low rate of variants even when it is in linkage disequilibrium (LD) with *HLA-A* [8]. Emphasizing the third point, *HLA-G* genetic variation affects its expression at the transcriptional and post-transcriptional level [9]; for that, it is important to explore the association between its genetic determinants and cancer susceptibility and progression [10].

The 3′ untranslated region (UTR) of the *HLA-G* gene contains signals that regulate the spatial and temporal expression of its mRNA [11]. It is notable that this region is polymorphic, which may impact the response to endogen cellular factors according to cellular type [12]. One of the most studied genetic variants in the 3′UTR of the *HLA-G* gene is rs66554220 [8,13], which produces a 14-bp insertion (Ins)/deletion (Del) of the sequence 5′-ATT TGT TCA TGC CT-3′ between the +2960 and +2961 position in exon 8 [14]. The Ins (wild-type) allele is associated with low *HLA-G* gene expression and low levels of free soluble HLA-G (sHLA-G) given the strong LD with other single-nucleotide variants (SNVs) [10]. On the other hand, the Del allele is related to an increased *HLA-G* gene expression and higher levels of sHLA-G [12,14].

A previously published meta-analysis suggested that the rs66554220 variant may not influence cancer susceptibility in an overall context [15,16]. Nevertheless, the role of *HLA-G* SNVs in BC has already been suggested based on their biological interactions, although their precise mechanisms of action remain unclear [17]. In this respect, the *HLA-G* gene rs66554220 variant has been studied in different populations, with discordant results regarding its association with BC [15,18,19]. In Mexican people, however, there are no published studies on its association with cancer.

Considering the aforementioned context and the lack of reports on other admixture populations such as Latin-Americans, we aimed to study the potential role of variant rs66554220 in the susceptibility and clinical outcome of BC in a Mexican population.

## 2. Materials and Methods

### 2.1. Subjects

We conducted a case-control study at Universidad de Guadalajara, in Jalisco, Mexico. The patient group included 182 Mexican women (due to the low incidence of breast cancer in men, only women were included) aged ≥18, diagnosed with de novo non-familial BC, clinically and histologically confirmed by medical oncologists and pathologists and recruited as a part of the earlier “ELLA Binational Breast Cancer Study” [20]. Their clinical features, including menopausal status; body mass index; molecular phenotype according to ER, PR and HER2/neu; clinical and pathological stage; and metastasis were obtained through medical records.

Also, we included a disease-free sex-age (±5 years)-matched reference group (sm-RG) which was composed of 221 healthy women, who, upon questioning, did not report having breast cancer; were randomly selected; without any history or laboratory evidence of infectious, heart-related, inflammatory and renal diseases; and without background of surgery or blood transfusions for at least one year at the time of sampling, with a mean age of 50.50 ± 11.43 years old. All participants were born in the state of Jalisco with ethnic ancestry of three generations from western Mexico and provided signed informed consent. The study was approved by the ethical and investigation committee from Universidad de Guadalajara (CI-9708) and conducted according to the Declaration of Helsinki, 1964.

### 2.2. Genotyping

Genomic DNA was obtained from peripheral blood using the salting-out method [21]. The rs66554220 variant was amplified by PCR using primer sequences modified from García-González et al., 2014 [22], to which two nucleotides (GT) were added at the beginning of the forward primer and one nucleotide (A) was added at the end of the reverse primer in order to adjust the two primers to the same alignment temperature, as follows: F: 5′-GTG ATG GGC TGT TTA AAG TGT CAC C-3′ and R: 5′-GGA AGG AAT GCA GTT CAG CAT GA-3′. The PCR reactions were performed using 20 ng of genomic DNA in a total volume of 10 µL, containing 1X PCR buffer, 1.5 mM MgCl_2_, 100 mM of each dNTP, 0.3 mM of each primer and 0.025 U of recombinant Taq DNA polymerase recombinant, all reagents from Invitrogen (Life Technologies Corporation, Carlsbad, CA, USA). Later, the reaction was carried out in a thermal cycler Aeris (Esco^®^ Lifesciences group, Changi, Singapore) with the following conditions: initial denaturation at 94 °C for 4 min; followed by 30 cycles of 26 s, each one at 94 °C, 65 °C and 72 °C; and final extension at 72 °C for 7 min. Fragments of 210 pb (Deletion) or 224 pb (Insertion) were obtained. These fragments were visualized in polyacrylamide gel electrophoresis (Golden Bell reagents, Jalisco, Mexico) at 6% in an OWL P9DS camera (Thermo Fisher Scientific, Waltham, MA, USA) and stained with silver nitrate (Golden Bell reagents, Jalisco, Mexico). As a quality control, 10% of all samples were selected, reanalyzed and all results were confirmed by an independent blinded observer.

### 2.3. Statistical Analysis

The allele and genotype frequencies were calculated via direct counting in both study groups. Hardy–Weinberg Equilibrium (HWE), χ^2^ and logistic regression were performed in the online SNPstats software: https://www.snpstats.net/ (accessed on 6 June 2023). Also, comparisons between allelic and genotypic frequencies vs. clinical characteristics in the BC group were made in IBM SPSS Statistics (v27.0). Values of *p* < 0.05 were considered significant. “Finally, the statistic power (1−b) was calculated in Post-hoc power calculator online (https://clincalc.com/Stats/Power.aspx, accessed on 6 June 2023) according to the sample size of the study, respectively”.

## 3. Results

### 3.1. Description of Clinical Variables

The clinical data of the BC patients are shown in Table 1. At the time of BC diagnosis, most of the patients were postmenopausal and 54.53 ± 12.53 years old. Moreover, according to their body mass index, most BC patients presented overweight (37.9%) or obesity (28.6%), and 33% of those were ≥60 years old. Regarding the molecular phenotype of cancer, Luminal A was the most predominant (29.1%). Additionally, the clinical and the pathological stage II were the most prevalent, with around 16% of metastasis reported.

### 3.2. Genetic Association

**Figure 1:**
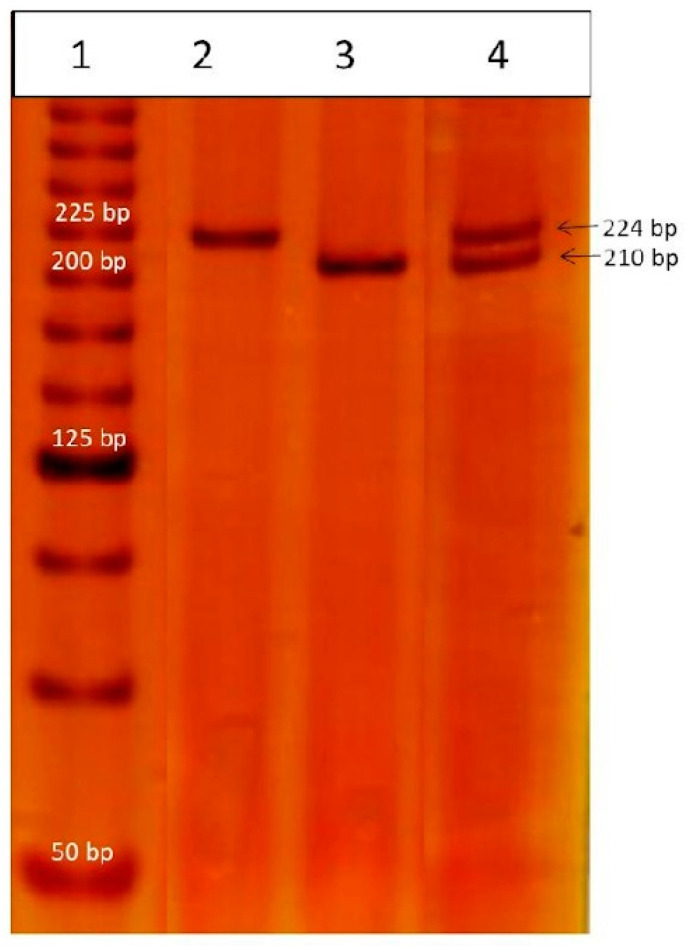
Visualization of *HLA-G* 14-bp Ins/Del (rs66554220) variant genotypes via 6% polyacrylamide gel electrophoresis. Lane 1, 25 base-pair molecular-weight markers indicating 125 bp as the most intense band; lane 2, homozygous Ins/Ins genotype; lane 3, homozygous Del/Del genotype; and lane 4, heterozygous Del/Ins genotype.

Allele and genotype frequencies for the rs66554220 variant were in agreement with HWE in BC patients (*p* = 0.61) and sm-RG (*p* = 0.79). Electrophoretic patterns of *HLA-G* 14-bp Ins/Del (rs66554220) variant genotypes are demonstrated in Figure 1. As shown in Table 2, according to χ^2^ and logistic regression with different inheritance models, no statistically significant differences were observed between groups. In addition to this, we stratified BC patients according to clinical features and we found the Ins allele as a possible risk factor for clinical stage IV in the BC group vs. the sm-RG (Table 3). However, the statistical power (41%) was insufficient to be a conclusive association. The remaining clinical features (menopausal status, body mass index, molecular phenotype, pathological stage and metastatic status showed no association).

## 4. Discussion

The *HLA-G* gene 3′UTR 14-bp (rs66554220) variant is involved in HLA-G production via modulating the mRNA stability of mechanisms that have not been yet fully elucidated [23,24], albeit the insertion of 14-bp (Ins) has been associated with low *HLA-G* mRNA production [23]. On the other hand, with the deletion of 14-bp (Del), the transcripts could be further processed by the removal of the first 92 bases of exon 8, producing a smaller and stable transcript as compared with the complete mRNA forms [23,24]. Nevertheless, it should be emphasized that the presence of the 14-bp insertion is always related to the presence of another two variants, rs1063320 and rs9382142 (+3142G and +3187A, respectively), in strong LD and is associated with a low quantity of *HLA-G* mRNA [25].

In the present study, genetic frequencies of the rs66554220 variant were not associated with BC, which is like other studies [26]. It is important to mention that the frequency of rs6655420 allele/genotypes was in accordance with earlier reports from the West of Mexico [22,27,28]. Furthermore, according to Farias-Rodrigues et al., the insertion allele has a similar distribution around the world, indicating a possible action of balancing selection [29]. This fact is important because HLA-G’s pivotal function in the immune system and its putative beneficial role in maintaining genetic variability in different stages of immunological processes [29]. In this way, it allows for a better understanding of the role of genetic variability in complex diseases such as BC.

**Figure 2:**
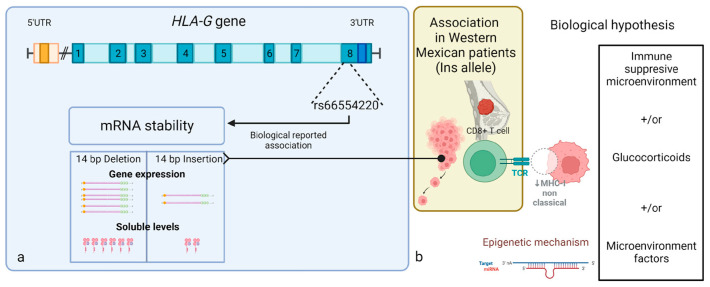
Biological hypothesis about risk association of Ins allele of rs66554220 in *HLA-G* gene with clinical stage IV breast cancer. (**a**) As a background, *HLA-G* gene contains 8 exons; their last exon, the 3′UTR, contains the rs66554220 variant related to mRNA stability given the Ins/Del of 14-bp associated with decreased/augmented gene expression and soluble levels of the HLA-G protein, respectively. (**b**) According to this research, we proposed the biological hypothesis derived from the clinical suggestion of association between Ins allele and clinical stage IV in patients from western Mexico with breast cancer as a risk factor, because their biological function is related to epigenetic mechanisms, an immune-suppressive microenvironment and other microenvironmental factors that allow the immune surveillance and evasion of the system MHC-I non classical. Created with BioRender.com.

Studies of different pathological conditions have indicated that the *HLA-G* gene might serve as a clinical marker for the diagnosis or prediction of the clinical outcomes of breast cancer [30,31,32,33,34]. In the present study, we suggest that the Ins allele could granted up to three times the risk of developing clinical stage IV BC, where the microenvironment changes from anti-tumor to tumor-promoting [2]. At this point, *HLA-G* gene expression can act as a checkpoint and as a critical marker of immune tolerance in cancer-cell immune evasion, disease progression and prognosis, given that the heterogeneity of their expression in immune-suppressive microenvironments and the isoform profiles vary among tumor type and patients [3] (Figure 2).

Our results are similar to the findings in South Indian women, in which the Ins allele is proposed as an important factor in the pathogenesis of BC. Nevertheless, they lacked an analysis of clinical variables [18]. Contrary to our findings, in populations from Tunisia and Iran, the Del allele is proposed as a risk factor for developing BC [19,32,33,34]. Also, in Brazilians, the Del/Del genotype is associated with higher levels of soluble HLA-G in invasive breast ductal carcinoma, poor prognosis of life and metastasis [35]. Moreover, in a recent meta-analysis in Caucasic and Asiatic populations, Tizaoui et al. suggested the rs66554220 variant as a risk factor and sHLA-G level as a biomarker for BC [6]. These similarities and differences remark the importance of studying different populations to ascertain the validity of a gene as a possible risk factor for a pathology, particularly in populations with high genetic admixture such as the Mexican population [36].

In the context of cancer, the immunoediting process includes the gain of expression of immune-inhibitory molecules such as HLA-G [37]. Furthermore, *HLA-G* gene expression can be induced by glucocorticoids or microenvironmental factors such as low oxygen tension or tryptophan starvation, both characteristic of cancer, along with it being regulated by epigenetic mechanisms [38]. Owing to the latter, the presence of the Ins allele could be an important regulator of the union of different microRNAs that allow HLA-G to function as a bipartite immune checkpoint, contributing in complex diseases such as BC.

The evaluation of the clinical characteristics such as menopause was exclusive for patients, and they were not comparable with the sm-RG; in addition, the stratification of patients based on their clinical characteristics reduced the statistic power to half of the statistical significance.

It is important to emphasize that this first approach is focused on the role of the rs66554220 variant in our population, and future case–control studies will be performed with other characteristics of women, with the inclusion of a larger number of patients to increase the robustness of what is proposed here. Also, future investigations with new methodologies will be needed. Finally, the prevalence of the clinicopathological stage and metastasis in our patients could be related to an early or late diagnosis more than a genetic factor in its entirety, taking into consideration that, in the Mexican population, the diagnostic rate of BC is above <50 years compared with the United States and Europe and it is also mostly in advanced stages (III, IV, N.C) in two out of three patients [39,40].

## 5. Conclusions

We concluded that the 14-bp Ins/Del (rs66554220) variant of *HLA-G* is not associated with BC in the Mexican population, but might be related to advanced breast tumors; further studies are required. We suggest the Ins allele as a possible risk factor for developing BC at clinical stage IV; nevertheless, a bigger stratified sample size might verify this. We propose the integration of clinical features in the association studies due to the possibility of identifying possible genetic factors involved in the etiopathogenesis of complex diseases like cancer and, as in other publications, we also suggest that the 3′UTR of the *HLA-G* gene segment should be analyzed in a wider approach because of its strong linkage disequilibrium with other variants. This could be useful in future clinical practice settings or in generating new strategies for the diagnosis–prognosis of cancer.

## Figures and Tables

**Table 1:** Clinical features of breast cancer patients.

**Modifiable Risk Factors**	**n (%)**
Menopausal status	
Pre-menopause	62 (34.1)
Post-menopause	118 (64.8)
Unknown	2 (1.1)
Body mass index	
Underweight	1 (0.5)
Normal weight	48 (26.4)
Overweight	69 (37.9)
Obesity class I	40 (22)
Obesity class II	6 (3.3)
Obesity class III	6 (3.3)
Unknown	12 (6.6)
**Mechanisms/Pathophysiology**	**n (%)**
Molecular phenotype	
Luminal A	53 (29.1)
Luminal B	22 (12.1)
HER2/neu	27 (14.9)
TNBC	23 (12.6)
Unknown	57 (31.3)
Clinical stage	
I	19 (10.4)
II	77 (42.3)
III	58 (31.9)
IV	11 (6.0)
Unknown	17 (9.4)
Pathological stage	
I	21 (11.5)
II	68 (37.4)
III	65 (35.7)
IV	9 (5)
Unknown	19 (10.4)
Metastatic status	
Presence	29 (15.9)
Absent	126 (69.2)
Unknown	27 (14.9)

TNBC, triple-negative breast cancer.

**Table 2:** Allelic and genotypic frequencies and the results of the association test of the *HLA-G* 14-bp Del/Ins (rs66554220) variant.

Inheritance Model	Sex-Matched Reference Group n = 221 (%)	Breast Cancer n = 182 (%)	*p*-Value *
Allele			
Del	(54)	(53)	Reference
Ins	(46)	(47)	n.s.
**Co-dominant**			
Del/Del	65 (29.4)	50 (27.5)	Reference
Del/Ins	108 (48.9)	94 (51.6)	n.s.
Ins/Ins	48 (21.7)	38 (20.9)	n.s.
**Dominant**			
Del/Del	65 (29.4)	50 (27.5)	Reference
Del/Ins + Ins/Ins	156 (70.6)	132 (72.5)	n.s.
**Recessive**			
Del/Del + Del/Ins	173 (78.3)	144 (79.1)	Reference
Ins/Ins	48 (21.7)	38 (20.9)	n.s.

*p*-value: significance defined by the χ^2^ test; n.s.: non-significant. * Due to the lack of statistical significance, the OR and 95% CI were omitted.

**Table 3:** Allelic frequencies and association test of the *HLA-G* 14-bp Del/Ins (rs66554220) variant in clinical stage IV of breast cancer clinical features.

Allele	Sex-Matched Reference Group n = 221 (%)	Clinical Stage IV of Breast Cancer n = 11 (%)	*p*-Value, OR (95% CI) *
Del	(54)	(27)	Reference
Ins	(46)	(73)	0.01, 3.05 (1.16–7.96)

*p*-value: significance defined by the χ2 test; OR: odds ratio; 95% CI: 95% confidence interval. * The statistic power (1−β) derived from this comparison was 41%.

## Data Availability

Research data are stored in our laboratory storage unit.
